# Silicate Dissolution Mechanism from Metakaolinite Using Density Functional Theory

**DOI:** 10.3390/nano13071196

**Published:** 2023-03-27

**Authors:** Mohammadreza Izadifar, Neven Ukrainczyk, Eduardus Koenders

**Affiliations:** Institute of Construction and Building Materials, Technical University of Darmstadt, Franziska-Braun-Str. 3, 64287 Darmstadt, Germany

**Keywords:** metakaolinite, density functional theory (DFT), improved dimer method, activation energy, dissolution

## Abstract

Metakaolin (MK) is a high-quality, reactive nanomaterial that holds promising potential for large-scale use in improving the sustainability of cement and concrete production. It can replace cement due to its pozzolanic reaction with calcium hydroxide and water to form cementitious compounds. Therefore, understanding the dissolution mechanism is crucial to fully comprehending its pozzolanic reactivity. In this study, we present an approach for computing the activation energies required for the dissolution of metakaolin (MK) silicate units at far-from-equilibrium conditions using the improved dimer method (IDM) and the transition-state theory (TST) within density functional theory (DFT). Four different models were prepared to calculate the activation energies required for breaking oxo-bridging bonds between silicate or aluminate units. Our results showed that the activation energy for breaking the oxo-bridging bond to a silicate neighbor is higher than that to an aluminate neighbor due to the ionic interaction. However, for complete silicate tetrahedra dissolution, a higher activation energy is required for breaking the oxo-bridging bond to the aluminate neighbor compared to the silicate neighbor. The findings provide methodology for missing input data to predict the mesoscopic dissolution rate, e.g., by the atomistic kinetic Monte Carlo (KMC) upscaling approach.

## 1. Introduction

Metakaolinite is a type of nanomaterial due to its small particle size, typically in the range of 10 to 100 nanometers, which gives it unique reactivity properties. It is a derivative of kaolinite that is produced by heating kaolinite to temperatures between 500 and 800 degrees Celsius, which causes it to undergo a phase transformation. All this makes it useful in various applications, such as in the production of cementitious, geopolymer, and catalyst materials. Its small particle size also makes it a suitable candidate for use in nanocomposites and nanofillers. In this paper, the focus is on reactivity of metakaolin in cement- and geopolymer-based materials, which involves the dissolution mechanism in alkaline pore solutions. The annual production of more than 20 billion tons of concrete contributes 6–8% to the worldwide anthropogenic carbon dioxide production [1]; in other words, 7% of all greenhouse gases released worldwide [2]. Nevertheless, Portland clinkers, which produce high levels of CO_2_ emissions, can be partially substituted with supplementary cementitious materials (SCMs) to reduce those emissions by 30–40% without substantially affecting the strength, durability, performance, or cost of the material [3]. Supplementary cementitious materials (SCMs) that are used in the cement industry generally comprise industrial waste products, natural pozzolans, and activated clay minerals that exhibit either pozzolanic or hydraulic properties. Kaolin calcination produces millions of tons of metakaolinite annually, which serves as a SCM, geopolymer precursor, and concrete nano-additive. The major mineral phase, “ideal or ordered kaolinite” (Al_2_(OH)_4_Si_2_O_5_), dehydroxylates into metakaolinite (Al_2_Si_2_O_7_) [4] at 500–700 °C (Figure 11 reported in [5]). “Low-crystallinity” or “disordered kaolinites” with stacking faults dehydroxylate into metadiskaolinite at lower temperatures (400–700 °C, Figure 12 reported in [5]). Complete dehydroxylation at stationary points causes metakaolinite and metadiskaolinite supercells to expand by around 4% and 8%, respectively, compared to ideal and disordered kaolinite, as shown in Figure 18 from the study reported by Izadifar et al. [5].

Garg and Skibsted’s recent study [6] measured the MK initial dissolution rate for 0.10 mol/L NaOH and longer-term reaction behavior. The initial dissolution rate remained constant at 38 µmol/L/h for both Al and Si for the first 24 h, in far-from-equilibrium (highly diluted) conditions. Subsequently, the dissolution rate slowed as it approached equilibrium, highlighting the need to distinguish the fundamental contributions of forward (and reverse) dissolution reactions from other precipitation reactions. Briki et al. [7] found that the slag reaction slowed in both the early and late stages. They observed a non-steady state dissolution stage for aluminum, silicon, and sulfur ions in the first 30 min of a slag-NaOH solution, followed by a steady stage and then a decrease in release rates for aluminum and silicon ions due to precipitation. The release rates of aluminum and silicon were initially identical but started to deviate after two days. Slag reactivity was six times higher in NaOH solution than in cement paste after one day, and the slower reaction in cement paste was attributed to the presence of calcium ions from clinker phases. On two kaolinite types, N’Guessan et al. [6] studied aluminum and silicon leaching in different alkali hydroxide solutions. Dissolution of kaolinites was related to the hydroxide concentration, with Na^+^ inducing more dissociation than K^+^ due to its higher charge density. Werling et al. [8] reported the amount of different phases formed during geopolymerization by determining the solubility of metakaolinite and amorphous SiO_2_ in NaOH solution at different concentrations. The increment of solubility of metakaolinite calcined at 700 °C has been observed with the increasing concentration of NaOH. In metakaolinite, solubilities of Si < 5% were reached after 24 h and even 7 days in 0.01 and 0.1 mol/L NaOH. In 10.79 mol/L NaOH, the solubilities of Si for the same metakaolinite reached 65% and 80% after 24 h and 7 days, respectively.

Atomistic computational methods have emerged recently as a powerful approach for understanding the microstructure of cement [9,10,11,12] and glasses [13] and their relationship to reactivity. Molecular dynamic (MD) [14,15,16], classical MD, density functional theory (DFT), and ab initio MD computations are extensively used in chemistry and materials science to investigate properties such as reaction mechanisms, mechanical properties computations [17,18], and activation and bonding energies by utilizing force fields or electronic density, respectively, to derive the system’s total energy. Kurganskaya and Luttge [19] used the kinetic Monte Carlo approach to study the dissolution of phyllosilicates and predict the temporal surface evolution dynamics from the kinetics of elementary surface reactions. Their findings suggested that the dissolution of these minerals occurs through the formation of etch pits at opened screw dislocation cores via the movement and coalescence of stepwaves. More recently, Izadifar et al. [20] investigated the nucleation of alkaline aluminosilicate gels based on the binding energies of four different monomer species, including dissolution of metakaolinite [21], using a coarse-grained Monte Carlo (CGMC) approach. However, they did not consider activation energies. Morrow et al. [22] investigated dissolution mechanisms for Al- and Si-terminated sites in protonated, neutral, and deprotonated states in aluminosilicate minerals using density functional theory calculations. Schliemann and Churakov [23,24] employed an ab initio meta-dynamics simulation approach to comprehensively investigate the atomistic scale mechanism for pyrophyllite dissolution on the (010) surface and at the (110) edge surface. Tian et al. [25] conducted DFT calculations on the crystalline and amorphous forms of the anorhite calcium-aluminosilicate. Finally, Salha et al. [26] demonstrated that Al substitution promotes alumino-silicate chain growth in neutral conditions. Their findings suggest that substituting Si with Al-IV, V, and VI units can lower the energy barriers for generating trimers and pentamers.

Despite extensive research in the field of clays, no atomistic computational approach has been reported, to date, for studying the reactivity of protonated silicate tetrahedra in metakaolinite (calcined) clay while accounting for the influence of the existing neighboring aluminate and silicate units. Nevertheless, DFT computational silicate chemistry has been well researched. Xiao et al. studied gas-phase hydrolysis of silicic acid dimer in the presence of OH^−^, finding that a 5-coordinated Si species forms with a 79 kJ/mol barrier, followed by dissociation into two silicic acid molecules with a 19 kJ/mol barrier. Pelmenschikov [27] found that Si–O–Si formation requires about 120 kJ/mol in the hydrolysis of silica in neutral and acidic solutions. Gong and White [13] used a force field molecular dynamics (MD) simulations approach to generate detailed structural representations for amorphous CaO-MgO-SiO2-Al_2_O_3_ and CaO-SiO_2_-Al_2_O_3_ glasses for their applications as SCMs in blended Portland cements. They have illustrated that the glass structural representations obtained through the MD simulation approach are in good agreement with the experimental results of X-ray and neutron pair distribution function data and literature data in terms of the nearest interatomic distance, coordination number, and degree of depolymerization.

After obtaining the atomistic activation energies as input data, Izadifar et al. examined dissolution of portlandite [11] and β-C_2_S clinker [12] through the atomistic kinetic Monte Karlo (KMC) upscaling approach [9,10]. However, the abovementioned aspects of the MK dissolution reaction mechanism have not been visited. Inspired by the pioneer works on silicates, we performed first-principles-based (DFT) calculations to highlight the mechanisms and kinetics for the MK dissolution, initially focusing on silicate-aluminate interactions in neutral aqueous solution. Hence, the main objective of this study is to compute the enthalpy activation energy (ΔH*) at far-from-equilibrium condition, which is based on the transition-state theory (TST) for the calculation of atomistic reaction rates for silicate tetrahedra dissolution in MK through the DFT computational approach. In general, the presented approach forms a basis for future DFT studies on aluminosilicate dissolution mechanisms. Thereby, our and future findings would provide missing data to feed mesoscopic forward dissolution rate computations, e.g., to run the atomistic kinetic Monte Karlo (KMC) upscaling approach.

## 2. Materials and Computational Approach

### 2.1. Structural Preparation

**Figure 1:**
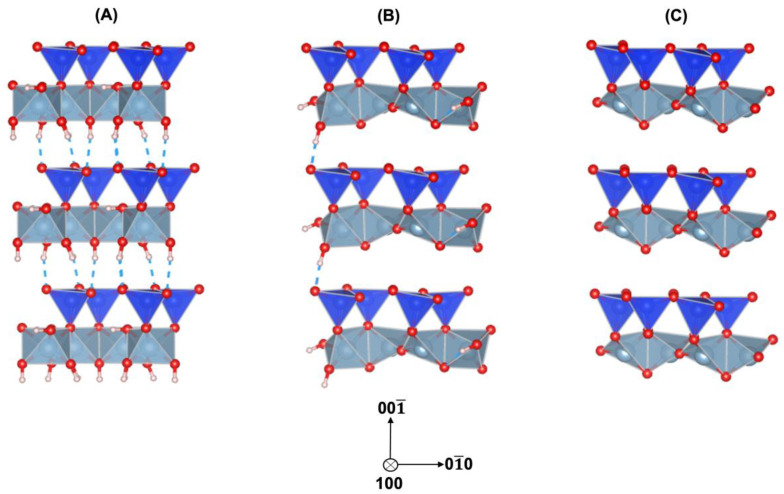
(**A**) The initial periodic 1 × 1 × 3 ideal kaolinite supercell, Al_12_Si_12_O_30_(OH)_24_. (**B**) Partially dehydroxylated ideal kaolinite, Al_12_Si_12_O_39_(OH)_6_. (**C**) Metakaolinite, Al_12_Si_12_O_42_. Silicate tetrahedrons are depicted in dark blue, aluminate octahedrons in light blue, oxygen atoms in red, and protons in white.

The initial periodic unit cell was expanded by a 1 × 1 × 3 ideal kaolinite supercell, as shown in Figure 1A. The unit cell parameters after expansion for B-vacant 1:1 layer were *a* = 5.15 Å, *b* = 8.94 Å, *c* = 22.18 Å, *α* = 91.92°, β = 105.04°, γ = 89.79°. Input structure was based on the transformation of ideal kaolinite Al_12_Si_12_O_30_(OH)_24_ into the partially dehydroxylated ideal kaolinite phase Al_12_Si_12_O_39_(OH)_6_ that took place after losing nine water molecules (Figure 1B), following Izadifar et al. [5], when the chemical potential of water was −1.93 eV. The final structure of the ideal MK implemented in this study is illustrated in Figure 1C. After losing three more water molecules, it had a water chemical potential of −2.67 eV and the chemical formula Al_12_Si_12_O_42_ with the space group P1.

### 2.2. Density Functional Theory (DFT) Calculation

The density functional theory (DFT) calculations [28] were carried out employing the Vienna ab initio simulation package (VASP) [29,30,31,32,33,34,35] with the projected-augmented wave (PAW) method [36,37] and pseudopotential were used to define electron-ion interaction. A well-converged plane-wave cutoff energy of 400 eV was employed for the structural relaxations. The electron exchange and correlation function were chosen in the generalized gradient approximation (GGA) with the Perdew-Burke-Ernzerhof (PBE) parametrization [38]. The Brillouin zone was sampled using a well-converged k-sampling equivalent given by 1 × 1 × 1 Monkhorst-Pack *k*-points mech size for the total system [39]. The break condition of 10^−5^ eV was set for the convergence criterion for the electronic self-consistent cycles. In these calculations, the ionic positions, cell volume and cell shape were relaxed until all the forces were smaller than 10^−3^ eV/Å. To ensure that the stress had also converged, a separate convergence criterion was employed for the stress. Specifically, the “STRESS-TENSOR” was required to reach a value of 0.01 kbar. A three-dimensional visualization software of (VESTA) was also utilized for the structural analysis of our models [40].

The intention for this study was to find the reaction transition states for silicate dissolution in MK through the dimer method using the DFT computational approach. In this way, and through the dimer method [41], the saddle point associated with the reaction and, consequently, the activation energy computation for silicate tetrahedra dissolution based on the transition state theory (TST) can be obtained. In fact, the dimer method is a technique for the optimization of the transition states at the atomic scale for the computation of a minimum energy pathway and finding the transition state between a reactant and a product state. In VASP calculation, the method has been improved by Heyden et al. [42] and, accordingly, it is called the improved dimer method (IDM). The minimum energy pathway represents how the atoms would evolve (between the given initial and the final states), and where a maximum in the potential energy along that path represents the activation energy of the studied process.

Initially, the individual geometries of the initial (reactants) and the final (products) systems are optimized to minimize their energy. Secondly, the initial dimer direction was provided, and then the vibrational modes of the TS were computed to find the mode of decay direction to be given in the improved dimer calculation (IDM). Finally, and through IDM, an optimization step was taken, and the potential energy was maximized along the unstable direction (i.e., dimer axis) while it was minimized in all other directions. It is also worth mentioning that the ground state coordination structure of all four presented models at the Transition state and final products have been provided in the supplementary materials.

## 3. Results and Discussions

**Figure 2:**
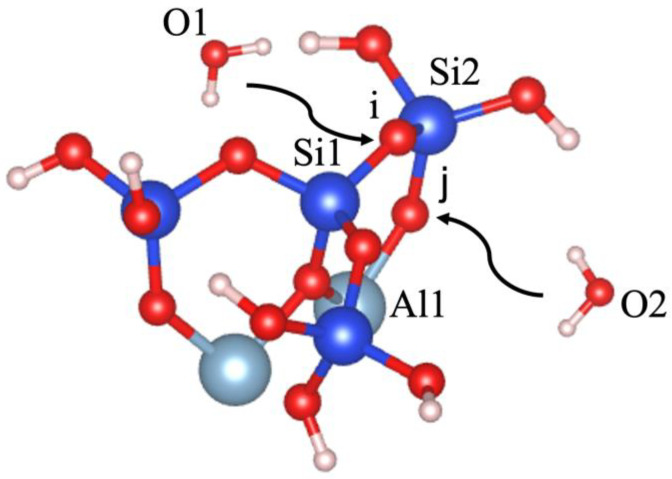
Illustration of the initial scenario for dissolution of silicate tetrahedra (Si(OH)_4_) depending on the bridging of oxygen to the silicate and aluminate units. Silicate atoms are shown in blue; aluminum in light blue; oxygen in red; and hydrogen protons in white.

**Figure 3:**
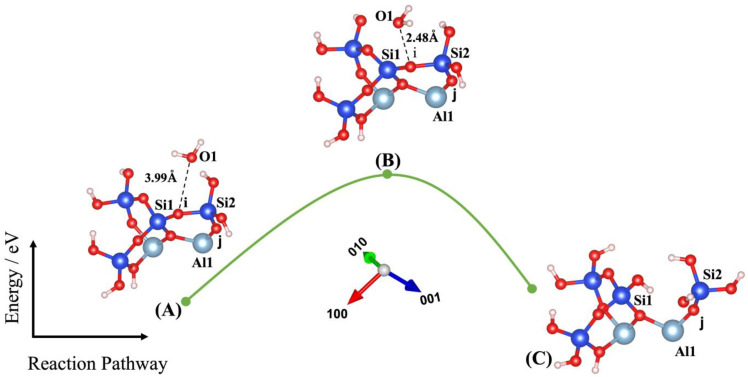
Model 1: (**A**) Ground state obtained as an optimized geometric structure of reactant, including water molecule as absorbent on MK surface (00$\overset{-}{1}$) for attacking and breaking the bridging oxygen, bonded to the silicate neighbor (Si1). (**B**) Transition state at the saddle point using improved dimer method. (**C**) Ground state obtained as an optimized geometric structure of product after protonation of the bridging oxygen to the silicate neighbor. The structural building units are described in caption of Figure 2. *E*_reactant, *E*_transition state, and *E*_product have been collected in Table 1.

**Figure 4:**
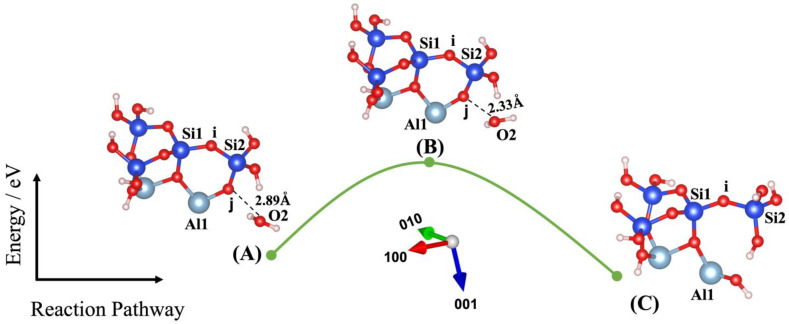
Model 2: (**A**) Ground state obtained as an optimized geometric structure of reactant, including water molecule as absorbent on MK surface (001) for breaking the bridging oxygen, bonded to the aluminum neighbor (Al1). (**B**) Transition state (TS) at the saddle point using improved dimer method. (**C**) Ground state obtained as an optimized geometric structure of product after protonation of the bridging oxygen, bonded to the aluminum neighbor. The structural building units are described in the caption of Figure 2. *E*_reactant, *E*_transition state, and *E*_product have been collected in Table 1.

**Figure 5:**
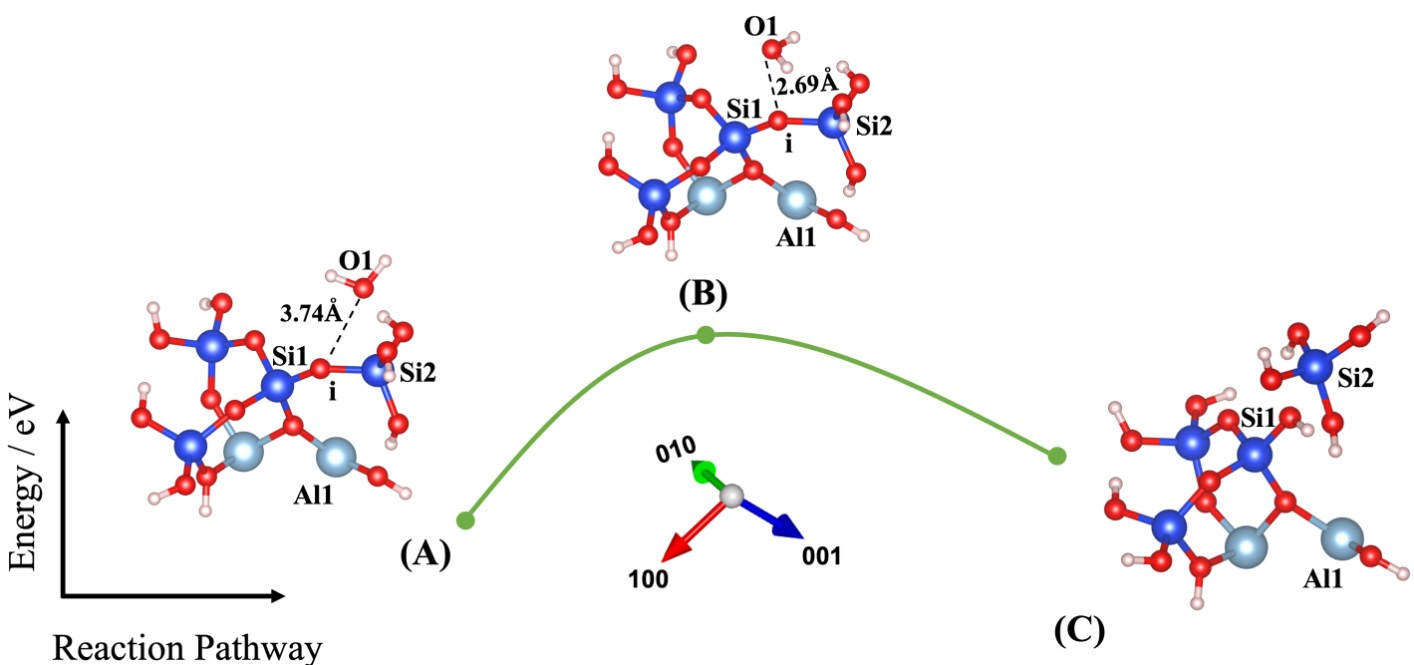
Model 3: (**A**) Ground state obtained as an optimized geometric structure of reactant, including water molecule as absorbent on MK surface (00$\overset{-}{1}$) obtained from model 2(C) for breaking the bridging oxygen, bonded to the aluminum neighbor (Al1). (**B**) TS at the saddle point using improved dimer method. (**C**) Ground state obtained as an optimized geometric structure of product after protonation of the bridging oxygen, bonded to the aluminum neighbor. The structural building units are described in caption of Figure 2. *E*_reactant, *E*_transition state, and *E*_product have been collected in Table 1.

**Figure 6:**
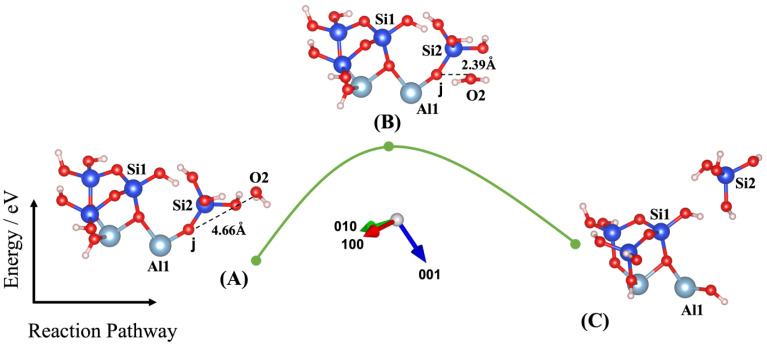
Model 4: (**A**) Ground state obtained as an optimized geometric structure of reactant including water molecule as absorbent on MK surface (001) obtained from model 1(C) for breaking the bridging oxygen, bonded to the silicate neighbor (Si1). (**B**) TS at the saddle point using improved dimer method. (**C**) Ground state obtained as an optimized geometric structure of product after protonation of the bridging oxygen, bonded to the silicate neighbor. The structure building units are described in caption of Figure 2. *E*_reactant, *E*_transition state, and *E*_product have been collected in Table 1.

Dissolution of silicate tetrahedra in MK depends on the different neighbors, representing different bonding via the bridging oxygen between silicate and aluminate unit neighbors, as shown in Figure 2. In fact, dissolution of silicate tetrahedra can be described through four various scenarios (models), depending on which bridging oxygen is hydrolyzed (bond broken and saturated by a proton). As illustrated in Figure 2, the first scenario can take place when the bridging oxygen (i) bonded to the silicate neighbor (Si1) is broken down by protonation through water molecule contribution (Figure 3). The second scenario involves protonation of the bridging oxygen (j) bonded to the neighboring aluminum (Al1) (Figure 4). The third scenario concerns silicate tetrahedra dissolution because of protonation of the bridging oxygen, bonded to the aluminum neighbor (Al1) (Figure 5) for the structure, which was obtained from the 1st scenario. The fourth scenario also concerns silicate tetrahedra dissolution by cause of protonation of the bridging oxygen, bonded to the silicate neighbor (Si1) (Figure 6), from the structure obtained from the 2nd scenario.

Liu et al. [43] have recently investigated the oligomerization (the reverse process of hydrolysis) of poly-silicic acid molecules, proceeding through the lateral attacking and simultaneous proton transfer from the approaching molecule for the formation of a 5-coordinated Si species as the transition state, resulting in the ejection of a water molecule from the formed poly-silicic acid. Therefore, we benchmarked our IDM with the results published by Liu et al., confirming the excellent repeatability of their results. Thus, our IDM implementation has been validated as an accurate method to evaluate the configuration of the structure and the computation of the total energy of the new (MK) system at the saddle point. For this, four different models (scenarios) have been identified in this study, as shown in Figure 3, Figure 4, Figure 5 and Figure 6. Findings are summarized in Table 1, listing for each scenario (model) the results of the (activation) energy barrier (*E_a_*) for the hydrolysis reaction, the energy change of the reaction enthalpy (*E_e_*), the O_water_-O_oxo_ distance between the attacking water and the oxo bridge, and the imaginary frequencies obtained from the first principles-based calculations for confirmation of the transition states. Moreover, all distances between the oxygen from water, Si, Al, and oxo-bridging in the reactants (A) and transition states (B) from Figure 3, Figure 4, Figure 5 and Figure 6 have been reported in Table 2 and Table 3, respectively.

The first scenario is representative of model 1, as shown in Figure 3. Figure 3A shows the ground-state structure of the reactant, including the water molecule, as absorbent with the distance of 3.99 Å (Table 2) from the bridging oxygen, bonded to the silicate neighbor (Si1) from the MK surface (00$\overset{-}{1}$). By performing IDM, as shown in Figure 3B, the water molecule rotated 90 degrees out of the plane and approached closer to the bridging oxygen at the distance of 2.48 Å (1.51 Å closer compared to the reactant structure). The activation energy was computed to be 1.753 eV as the energy of the system reached from −178.155 eV for reactant structure to the −176.402 eV for the TS structure. Figure 3C shows that the ground state obtained as an optimized geometric structure of the product was a result of protonation of the bridging oxygen through proton transfer from water and making silicate tetrahedra configuration (4-coordinated Si) for the neighboring silicate by contribution of the hydroxyl group from water.

The second scenario is presented as model 2 (Figure 4), in which it is considered that water initially attacks the bridging oxygen bonded to the aluminum neighbor on the MK surface (001). For the ground-state structure of the reactant, the water molecule is initialized within 2.89 Å from the bridging oxygen, bonded to the aluminum neighbor (Al1) from the MK surface 001, as shown in Figure 4A. After implementation of IDM, water rotated slightly and approached closer to the oxygen bridging, bonded to the aluminum neighbor (Al1) at the distance of 2.33 Å.

The activation energy of 0.891 eV was computed when the energy of the system reached from −177.920 eV for the reactant structure to −177.029 eV for the TS structure. As can be observed from Figure 4C, the ground state was obtained as an optimized geometric structure of the product as a result of protonation of bridging oxygen through proton transfer from water and making silicate tetrahedra configuration (4-coordinated Si) for the neighboring silicate by contribution of a hydroxyl group from water. After protonation of the initial oxo-bridging, depending on the silicate (Si1) or aluminum (Al1) neighbor for two different scenarios represented as models 1 and 2, models 3 and 4 illustrate the silicate tetrahedra dissolution, depending on the protonation of oxo-bridging, also bonded to the silicate or aluminate neighbor units. Model 2(C), with one water molecule involvement, is used as the initial structure for the third scenario, presented as model 3, when the water molecule attacks the bridging oxygen, bonded to the silicate neighbor (Si1), for silicate tetrahedra dissolution on the surface (00$\overset{-}{1}$), as shown in Figure 5.

Figure 5A shows the ground-state structure of the reactant presented as model 3 (obtained from model 2(C), as shown in Figure 4), including the water molecule as absorbent, with the distance of 3.74 Å from bridging oxygen, bonded to the silicate neighbor (Si1). When the IDM was implemented, the water molecule rotated almost 90 degrees and came closer to the bridging oxygen at the distance of 2.69 Å. The activation energy for model 3 was equal to 1.420 eV as the energy of the system reached from −192.843 eV for the reactant structure to −191.423 eV for the TS structure. Figure 5C shows the ground-state structure of the product, when the silicate tetrahedra dissolved after complete protonation of oxo-bridging. Concerning the fourth and the last scenario for dissolution of silicate tetrahedra, model 1(C) with the contribution of one water molecule was used as the initial structure (the reactant) for model 4, when the water molecule attacks the bridging oxygen bonded to the aluminum neighbor (Al1) on surface (001) as illustrated in Figure 6. Figure 6A displays that 4.66 Å was computed for the distance between water and bridging oxygen, bonded to the silicate neighbor (Si1) for the ground-state structure of the reactant.

After running the IDM optimization for model 4 (Figure 6), the water molecule reaches very close to the bridging oxygen at the distance of 2.39 Å. The computed activation energy of 1.641 eV was obtained as the energy difference between the reactant system structure (−192.664 eV) and the transition-state structure (*E*_TS_ = −191.023 eV). The final configuration of Figure 6C is identical with that of Figure 5C for complete dissolution of silicate tetrahedra, representative of the ground-state structure of the product. The findings of this study can be discussed by the following points. The activation energy needed to initiate the reaction concerning model 1 for breaking the oxo-bridging bonded to the silicate neighbor is 0.862 eV (97%) higher than that of the aluminum neighbor through the ionic interaction (model 2). On the contrary, for complete silicate tetrahedra dissolution, 0.221 eV (13%) higher activation energy is needed for the breaking of the oxo-bridging, bonded through the ionic interaction to the aluminum neighbor (model 4), in comparison to the breaking of oxo-bridging, bonded through the covalent bond to the silicate neighbor (model 3). Moreover, model 3 for complete silicate tetrahedra dissolution needs 0.333 eV (23%) less activation energy in comparison to model 1 for protonation of oxo-bridging, bonded to the silicate neighbor. In contrast, model 4 for complete silicate tetrahedra dissolution needs 0.750 eV (84%) more activation energy in comparison to model 2 for protonation of the initial oxo-bridging, bonded to the aluminum neighbor.

Based on the analysis of activation energies, it can be concluded that breaking the initial oxo-bridge with an Al neighbor, as shown in model 2, requires almost half the energy (85.96 kJ/mol) compared to the other presented models, namely 1 (169.1 kJ/mol), 3 (137.009 kJ/mol), and 4 (158.33 kJ/mol). Additionally, the highest activation energy was observed in the breaking of the initial oxo-bridge with a Si neighbor, due to covalent bonding.

Comparing our results with previous studies, Chen and Brantley [44] reported almost the identical activation energy values of 88.9 ± 14.6, and 85.2 kJ/mol for the dissolution of albite at acidic and basic pH, respectively, when breaking the initial oxo-bridge with an Al neighbor, as presented in model 2. Morrow et al. [22] reported a lower ab initio barrier of 79 kJ/mol for breaking of Si-O-Al in the deprotonated state of aluminosilicate mineral.

Furthermore, Nangia and Garrison [45] computed values of 110, 159 (which is almost identical to our computation), and 69 kJ/mol for breaking of Si–O–Si in deprotonated, neutral, and protonated states of aluminosilicate mineral, respectively. Pelmenschikov et al. [27] reported that hydrolysis of the first Si–O–Si bond in a cluster with four Si–O–Si links requires a higher value of 36 kJ/mol (205.20 kJ/mol) in comparison to our computation result. It was also noted that a value of 73.34 kJ/mol is needed to break the last remaining bond.

In general, the presented approach forms a basis for future DFT studies on aluminosilicate dissolution mechanisms, identifying all other possible scenarios needed to provide a comprehensive database for upscaling the dissolution rate simulations, e.g., by developing atomistic kinetic Monte Carlo (KMC) models.

## 4. Conclusions

In this paper, an improved dimer method (IDM) for optimization of the transition states through a density functional theory (DFT) computational approach was employed to study silicate dissolution of metakaolin in water. For this, four different models (scenarios) have been identified to calculate the activation energies of breaking oxo-bridging, depending on the silicate or aluminate neighbor arrangements. Based on the results of this study, the following conclusions could be drawn.

The activation energy for breaking the oxo-bridging covalent bond between the two silicates is 97% higher than the ionic bond between the silicate and aluminate units. However, for final (complete) silicate tetrahedra dissociation, 13% higher activation energy is needed for the ionic bond with the (last) alumina neighbor compared to the covalent bond with the (last) silicate neighbor. In addition, 23% less activation energy is needed for model 3, for complete silicate tetrahedra dissociation, compared to model 1, for the protonation of the covalent bond between the two silicates. Nevertheless, for complete silicate tetrahedra dissociation, model 4 needs 84% more activation energy compared to model 2 for protonation of the initial oxo-bridging bond with the alumina.

The approach demonstrated in this paper is fundamental for computing activation energies, which could be used, e.g., for mesoscopic dissolution rates using the atomistic kinetic Monte Carlo (KMC) upscaling approach, to link meta-clay crystallographic structure with dissolution reactivity.

## Figures and Tables

**Table 1:** Results for each scenario (model): energy barrier for the hydrolysis reaction (Δ*E_a_* = *E*_transition state − *E*_reactant), the energy change of reaction enthalpy (Δ*E_e_* = *E*_product − *E*_reactant), the Owater–Ooxo distance between the attacking water and the oxo bridge, and the imaginary frequencies obtained from first principles-based calculations for confirmation of the transition states.

Models	Δ*E_a_* (eV)	Δ*E_a_* (kJ/mol)	*E*_Reactant (eV)	*E*_Transition State (eV)	*E*_Product (eV)	Δ*E_e_* (eV)	O_water_–O_oxo_ Distance (Transition State) (Å)	Imaginary Frequencies (THz)
(1)	1.753	169.138	−178.155	−176.402	−177.983	0.172	2.480	−74.480
(2)	0.891	85.968	−177.920	−177.029	−178.044	−0.124	2.330	−74.106
(3)	1.420	137.009	−192.843	−191.423	−191.900	0.943	2.690	−76.330
(4)	1.641	158.330	−192.664	−191.023	−191.900	0.764	2.390	−81.593

**Table 2:** The distances between the oxygen from water, Si, Al, and oxo-bridging in the reactants (A), as shown in Figure 3, Figure 4, Figure 5 and Figure 6.

Models	O1-i (Å)	O1-Si1 (Å)	O1-Si2 (Å)	Si1-i (Å)	Si2-i (Å)	O2-j (Å)	O2-Si1 (Å)	O2-Si2 (Å)	O2-Al1 (Å)	Al1-j (Å)	Si2-j (Å)
(1)	3.99	4.23	3.79	1.61	1.67	-	-	-	-	1.76	1.62
(2)	-	-	-	1.62	1.66	2.89	5.58	3.39	4.02	1.78	1.64
(3)	3.74	4.09	3.86	1.60	1.66	-	-	-	-	-	-
(4)	-	-	-	-	-	4.66	6.35	3.37	5.97	1.75	1.61

**Table 3:** The distances between the oxygen from water, Si, Al, and oxo-bridging in the transition states (B) as shown in Figure 3, Figure 4, Figure 5 and Figure 6.

Models	O1-i (Å)	O1-Si1 (Å)	O1-Si2 (Å)	Si1-i (Å)	Si2-i (Å)	O2-j (Å)	O2-Si1 (Å)	O2-Si2 (Å)	O2-Al1 (Å)	Al1-j (Å)	Si2-j (Å)
(1)	2.48	2.54	3.15	1.65	1.67	-	-	-	-	1.78	1.62
(2)	-	-	-	1.61	1.66	2.33	5.09	2.79	3.44	1.74	1.63
(3)	2.69	2.89	3.49	1.64	1.67	-	-	-	-	-	-
(4)	-	-	-	-	-	2.39	5.11	2.16	3.33	1.79	1.69

## Data Availability

The data presented in this study are available on request from the corresponding authors.

## Supplementary Materials

The following supporting information can be downloaded at: https://www.mdpi.com/article/10.3390/nano13071196/s1.
